# Outer Membrane Vesicles Mediate Transport of Biologically Active *Vibrio cholerae* Cytolysin (VCC) from *V. cholerae* Strains

**DOI:** 10.1371/journal.pone.0106731

**Published:** 2014-09-04

**Authors:** Sridhar Elluri, Constance Enow, Svitlana Vdovikova, Pramod K. Rompikuntal, Mitesh Dongre, Sven Carlsson, Amit Pal, Bernt Eric Uhlin, Sun Nyunt Wai

**Affiliations:** 1 Department of Molecular Biology, The Laboratory for Molecular Infection Medicine Sweden (MIMS), Umeå University, Umeå, Sweden; 2 Division of Pathophysiology, National Institute of Cholera and Enteric Diseases, Kolkata, West Bengal, India; 3 Department of Medical Biochemistry and Biophysics, Umeå University, Umeå, Sweden; University of Alberta, Canada

## Abstract

**Background:**

Outer membrane vesicles (OMVs) released from Gram-negative bacteria can serve as vehicles for the translocation of virulence factors. *Vibrio cholerae* produce OMVs but their putative role in translocation of effectors involved in pathogenesis has not been well elucidated. The *V. cholerae* cytolysin (VCC), is a pore-forming toxin that lyses target eukaryotic cells by forming transmembrane oligomeric β-barrel channels. It is considered a potent toxin that contributes to *V. cholerae* pathogenesis. The mechanisms involved in the secretion and delivery of the VCC have not been extensively studied.

**Methodology/Principal Findings:**

OMVs from *V. cholerae* strains were isolated and purified using a differential centrifugation procedure and Optiprep centrifugation. The ultrastructure and the contents of OMVs were examined under the electron microscope and by immunoblot analyses respectively. We demonstrated that VCC from *V. cholerae* strain V:5/04 was secreted in association with OMVs and the release of VCC via OMVs is a common feature among *V. cholerae* strains. The biological activity of OMV-associated VCC was investigated using contact hemolytic assay and epithelial cell cytotoxicity test. It showed toxic activity on both red blood cells and epithelial cells. Our results indicate that the OMVs architecture might play a role in stability of VCC and thereby can enhance its biological activities in comparison with the free secreted VCC. Furthermore, we tested the role of OMV-associated VCC in host cell autophagy signalling using confocal microscopy and immunoblot analysis. We observed that OMV-associated VCC triggered an autophagy response in the target cell and our findings demonstrated for the first time that autophagy may operate as a cellular defence mechanism against an OMV-associated bacterial virulence factor.

**Conclusion/Significance:**

Biological assays of OMVs from the *V. cholerae* strain V:5/04 demonstrated that OMV-associated VCC is indeed biologically active and induces toxicity on mammalian cells and furthermore can induce autophagy.

## Introduction

*Vibrio cholerae* is considered a deadly pathogen that still poses a major health threat in developing and underdeveloped regions of the world [1]. *V. cholerae* pathogenesis has been primarily attributed to expression of two major virulence factors, cholera toxin (CT) and the toxin-coregulated pilus (TCP) [2]. These major virulence factors are expressed by only two serogroups of *V.* *cholerae*, namely O1 and O139, which are the only serogroups known to cause epidemic cholera outbreaks [3]. A vast majority of other *V. cholerae* serogroups, collectively termed the non-O1, non-O139 (NOVC) serogroups, lack CT and TCP and are therefore considered non-pathogenic [4], [5]. Recent decades, however, have seen an upsurge in sporadic outbreaks of non-cholera-causing *V.* *cholerae* infections involving NOVC strains [6]. In addition, naturally occurring CT-deficient *V. cholerae* O1 strains have also been reported to exhibit virulence in humans [7], [8]. Interestingly, in contrast to O1 and O139 serogroups, the infections caused by NOVC strains are mostly extra-intestinal infections such as cellulitis, meningitis, wound infections, septicemia and bacteremia [2], [9], [10].

Clinical NOVC isolates can express a variety of accessory virulence factors such as heat-stable enterotoxin (NAG-ST), CT-like enterotoxin, Shiga-like toxin, zonula occludens toxin (ZOT), repeats in toxin (RTX), cytolysin or hemolysin (VCC or HlyA), hemagglutinin protease (HapA), among others, which can be implicated in their toxigenic potential [2], [9]. While most of these putative virulence factors are expressed in only a fraction of clinical NOVC isolates, RTX, HapA and VCC are expressed in almost all *V. cholerae* serogroups and are therefore more promising as potential toxigenic factors [2], [9], [11]. VCC is a particularly interesting candidate; in a study using a streptomycin-fed murine model of disease, VCC was shown to be the predominant cause of death [12]. Moreover, studies employing the rabbit ileal loop model and human intestinal epithelium cell lines further established the pathogenic significance of VCC. In these studies, VCC induced enterotoxicity and apoptosis during *V.* *cholerae* infection, in contrast to cholera toxin [13], [14].

*V. cholerae* cytolysin, encoded by the *vca0219* gene, is a water-soluble, pore-forming toxin secreted as a 79-kDa inactive pro-cytolysin or pro-hemolysin that undergoes a post-translational N-terminal cleavage, mediated mainly by HapA and other proteases of *V. cholerae* to form an active, 65-kDa toxin [15]–[17]. Mature VCC forms heptameric channels upon contact with cholesterol- and ceramide-rich membranes [18], [19]. In in vitro experiments, culture supernatants as well as purified VCC were cytolytic to a wide variety of erythrocytes, and caused extensive vacuolization of different mammalian cells, depending on the cell line and toxin concentration [20], [21].

Recently, Gutierrez et al. reported that VCC could induce autophagy in CHO cells [22]. Autophagy is an intracellular mechanism of self-degradation of cellular components that serves an important role in cellular homeostasis, starvation adaptation, development, anti-aging, elimination of pathogens, cell death, and tumor suppression [23]. This evolutionarily conserved process results in the formation of a double-membraned autophagosome that engulfs cytoplasmic components and delivers them to the lysosome. Autophagy can be triggered by different signaling pathways involving a variety of regulators and autophagy-related genes (Atg). One such cellular factor is microtubule-associated protein 1 light chain 3 alpha (LC3), a mammalian homologue of Atg8, which is expressed on the inner membrane of autophagosomes and is frequently used for monitoring autophagy progression [24]. Recent reports also suggested active involvement of autophagy in cellular defense against apparent destructive effects of some secreted bacterial toxins, including VCC [22].

*V. cholerae*, like many other Gram-negative pathogens, employs different secretion pathways for effective delivery of specific virulence factors. *V. cholerae* constitutively releases outer membrane vesicles (OMVs), which can serve as safe delivery vectors for contact-independent targeted delivery of specific toxins to distant host cells [25], [26]. Notable examples of OMV-delivered toxins in other bacteria include, ClyA of *E. coli* [26], heat-labile toxin of Enterotoxigenic *E. coli* (ETEC) [27], cytolethal distending toxin of *Campylobacter jejuni* [28], leukotoxin and cytolethal distending toxin of *Actinobacillus actinomycetemcomitans* [29], [30], and VacA of *Helicobacter pylori* [31]. In this study, we aimed to elucidate the specific *V. cholerae* protein export machinery involved in VCC secretion by tracking intra- and extra-cellular translocation of VCC. In addition, we examined a possible role for *V. cholerae* OMVs in VCC secretion.

## Materials and Methods

### Ethics Statement

This study was carried out in accordance with the recommendations of the local ethical committee (Regionala etikprövningsnämnden i Umeå). Ethics committee approval for use of rabbit red blood cells for this study was issued on May 26, 2012 for a three year period with the permit number Dnr: A76-12.

### Bacterial strains, culture conditions and plasmids

The bacterial strains and plasmids used in this study are listed in Table 1. *V. cholerae* strains were grown overnight (O/N) under shaking conditions at 37°C either in Luria-Bertani (LB) broth or in Brain Heart Infusion (BHI) broth, whereas *E. coli* strains were grown until OD600 = 2.0 in Luria-Bertani (LB) broth. Carbenicillin (Cb) 50 µg/ml was used whenever necessary.

**Table 1:** Bacterial strains and plasmids.

Strains/Plasmids	Relevant genotype or phenotype	Source or reference
**Bacteria**		
*V. cholerae* V:5/04	non-O1 non-O139 clinical isolate (2004)	Swedish Institute of Infectious Diseases, Sweden
*V. cholerae* V:5/04Δ*vcc*	Δ*vcc* derivative of V:5/04	This study
*V. cholerae* V:5/04/pMMB66EH	V:5/04 carrying pMMB66EH vector	This study
*V. cholerae* V:1/05	non-O1 non-O139 clinical isolate (2005)	Swedish Institute of Infectious Diseases, Sweden
*V. cholerae* V:7/04	non-O1 non-O139 environmental isolate (2004)	Swedish Institute of Infectious Diseases, Sweden
*V. cholerae* V:11/04	non-O1 non-O139 clinical isolate (2004)	Swedish Institute of Infectious Diseases, Sweden
*V. cholerae* WO6	O1 clinical isolate	[64]
*E. coli* DH5α	F^−^, ø80d*lacZ*ΔM15, Δ(*lacZYA-argF*) U169, *deoR*, *recA*1, *endA*1,*hsdR*17 (rk^−^, mk^+^), *phoA*, *supE*44, λ^−^, *thi*-1, *gyrA*96, *relA*1	[65]
*E. coli* SM10λpir	*thi thr leu tonA lacY supE recA*::RP4-2 Tc::Mu Km λpir	[66]
*E. coli* MC1061	*araD139* Δ*(ara, leu)* 7697 Δ*lac*X74 *galU galK hsr hsm*+ *strA*	[67]
*E. coli* MC1061/pBR322	MC1061 with pBR322 vector	This study
*E. coli* MC1061/pBAB	MC1061 expressing VCC	This study
**Plasmids**		
pCVD442	Cb^R^ positive selection suicide vector plasmid	[68]
pCVD442::Δ*vcc*	pCVD442-based suicide plasmid for generating Δ*vcc*	[32]
pMMB66EH	P*tac* expression vector; Ap^r^	[69]
pBR322	Cb^R^ cloning vector plasmid	[70]
pBAB2	pBR322-based VCC expression plasmid	This study

### Media for cell lines and culture conditions

HeLa cells (ATCC CCL-2) were cultured in advanced minimum essential medium (AMEM), whereas HEK293 cells over-expressing GFP-fused LC3 were cultured in DMEM containing high glucose (GlutaMAX) at 37°C in a 5% CO_2_ incubator. For all experiments, culture media was supplemented with 2 mM glutamine, 10% FBS, 100 units/ml of penicillin and 100 µg/ml of streptomycin (Gibco). Fifty µg/ml gentamicin was added prior to the treatment with OMVs.

### Construction of vcc mutant strain

A Δ*vcc* mutant of V:5/04 strain was constructed by crossover PCR essentially as described previously [32]–[35].

### Sub-cellular fractionation

Sub-cellular fractionation was performed as described earlier [28]. For the whole cell lysate fractions, bacteria (500 µl) from the cultures (OD600 = 2.0) were centrifuged at 12,000×g for 5 min and re-suspended in 100 µl of SDS-PAGE sample buffer. Periplasmic proteins were isolated by osmotic shock as follows. The bacteria were washed with 20 mM Tris-HCl pH 8.0 and resuspended in 0.25 volume (compared to the starting volume) of a solution containing 20% sucrose, 20 mM Tris-HCl pH 8.0, and 1 mM Na-EDTA. The mixture was incubated for 10 min at room temperature. Subsequently, the bacteria were pelleted and resuspended in one volume of ice-cold 0.5 mM MgCl_2_. After incubation on ice for 10 min, the cells were removed by centrifugation at 12,000×g. The supernatant was filtered through a 0.22-µm syringe filter and used as the periplasmic protein extract. The cell pellet was then disrupted by sonication in one volume of 10 mM Tris-HCl pH 8.0 buffer. The cell debris and unbroken cells were removed by centrifugation at 5,000×g for 10 min at 4°C and the supernatant was filtered through a 0.22-µm syringe filter and subsequently centrifuged at 10,000×g for 30 min at 4°C. The supernatant was used as the cytoplasmic fraction. The sediment was resuspended with sterilized distilled water and used as the membrane fraction. In order to separate the inner and outer membranes, the fraction was further treated with N-lauryl sarcosyl at a final concentration of 2% at room temperature and then centrifuged at 15,000×g for 30 min. The resulting sediment was resuspended in 50 µl of sample buffer (50 mM Tris-HCl pH 6.8, 10% glycerol, 5% β-mercaptoethanol, 2% sodium dodecyl sulfate [SDS], 0.05% bromophenol blue) and used as the outer membrane fraction. The supernatant was used as the inner membrane fraction after dialysis and it was concentrated by precipitation. Extracellular, periplasmic, cytoplasmic and inner membrane fractions were concentrated by precipitation with ice-cold trichloroacetic acid (final concentration, 10%). The precipitated proteins were collected by centrifugation at 12,000×g, washed with acetone, dried under vacuum, and dissolved in 50 µl of same sample buffer that was used for outer membrane sample preparation above. Samples were neutralized by addition of saturated Tris solution and boiled for 5 min at 100°C. Five microliters of each fraction were loaded in the wells for SDS-polyacrylamide gel electrophoresis (SDS-PAGE) and immunoblot analysis of proteins. We estimated the amount of VCC in the OMVs by immunoblot analysis using known amounts of purified VCC as reference. 100 µl of our OMVs samples from the wild type *V. cholerae* strain V:5/04 contained an amount of VCC protein that was equivalent to 0.3 nmole of purified VCC. SDS-PAGE and immunoblot analysis were performed as described previously [36], [37]. Crp or RpoS, ToxR, β-lactamase and OmpU were used as cytoplasmic, inner membrane, periplasmic, and outer membrane localization markers respectively.

### Isolation and fractionation of OMVs

OMVs were isolated from *V. cholerae* and *E. coli* strains as described [26]. In brief, culture supernatants were obtained by centrifuging the bacterial cultures at 5000×g for 15 min at 4°C. The centrifugation step was repeated twice to remove residual amounts of bacteria. To avoid contamination with bacteria, the supernatants were sequentially filtered through 0.45-µm and 0.22-µm syringe filters. The bacteria-free supernatants were then centrifuged at 100,000×g at 4°C for 2 h and 3 h respectively for *V. cholerae* and *E. coli* in a 45 Ti rotor (Beckman Instruments, Inc.). The pellets were suspended in 20 mM Tris-HCl pH 8.0 or in Phosphate buffered Saline (PBS) and used as the OMV preparation. The protein content of the isolated OMVs was estimated using a Bicinchoninic Acid (BCA) Assay kit (Thermo Scientific Pierce, Rockford, IL). These OMV preparations were fractionated by density gradient centrifugation essentially as described earlier [29], [38]. In brief, the OMVs were isolated as described above and suspended in 20 mM Tris-HCl (pH 8.0) adjusted to 45% Optiprep (Sigma-Aldrich) in a final volume of 400 µl and transferred to the bottom of a 4-ml ultracentrifugation tube, and then different Optiprep/Tris-HCl layers were sequentially added as follows: 0.6 ml 35%, 0.6 ml 30%, 0.6 ml 25%, 0.6 ml 20%, 0.5 ml 15% and 0.4 ml 10%. Gradients were centrifuged (180, 000×*g*, 3 h, 4°C) in a SW60Ti rotor (Beckman Instruments Inc.), and fractions of equal volumes (300 µl) were sequentially removed and analyzed by immunoblot using anti-VCC and anti-OmpU antibodies.

### Dissociation assay

OMV samples (100 µg of total protein) in 20 mM Tris-HCl pH 8.0 were incubated on ice for 1 h in the absence or presence of either NaCl (1 M), Na_2_CO3 (0.1 M) pH 10.0, Urea (0.8 M) or 1% SDS [28], [38]. Samples were then centrifuged at 100,000×*g* for 2 h at 4°C and both pellet and supernatant fractions were analyzed by immunoblot using anti-VCC polyclonal antiserum. Before loading, the soluble proteins in the supernatant were concentrated by TCA-precipitation.

### Proteinase K susceptibility assay

The proteinase K susceptibility assay was carried out as previously described [39]. Briefly, OMVs isolated from the *V.* *cholerae* strain V:5/04 were treated with proteinase K (1 µg ml^−1^ ) in 20 mM Tris-HCl (pH 8.0) at 37°C for 30 min in the absence or presence of 1% SDS. Subsequently, the samples were analyzed by immunoblot analysis using anti-VCC antiserum.

### Electron microscopy and immunogold labeling

OMV preparations were negatively stained with a solution of 0.1% uranyl acetate on carbon coated Formvar grids and examined under the electron microscope. Micrographs were taken with a JEOL 2000EX electron microscope (JEOL Co., Ltd., Akishima, Japan) operated at an accelerating voltage of 100 kV [40].

For immunoelectron microscopy, a colloidal gold probe (Wako Pure Chemical Industries Ltd., Osaka, Japan) was used to label the specific reaction sites of anti-VCC serum in the specimens of OMVs from the wild type *V. cholerae* strain V:5/04 and its Δ*vcc* mutant. To label the specimens, a 50 µl sample (ca 3 µg protein) of the OMV preparation was treated with antiserum appropriately diluted in PBS for 30 min at 37°C. The OMVs then were separated from the serum by centrifugation at 100,000×g for 2 h at 4°C. After being washed three times with PBS, the OMV samples were mixed with a suspension of the colloidal gold probe, and the mixture was kept at room temperature for 30 min. After being washed with PBS to remove unbound gold particles, the OMV samples were negatively stained with 0.1% uranyl acetate on carbon coated Formvar grids and examined under the electron microscope.

### SDS-PAGE and immunoblot analysis

SDS-PAGE and immunoblotting was performed as described earlier [36], [37], [41]. In brief, the whole cell extracts, different sub-cellular fractions, supernatant fractions, and the isolated OMVs were subjected to polyacrylamide gel electrophoresis and then blotted onto a PVDF membrane. Immunoblot analyses were performed using anti-VCC polyclonal antiserum (1∶10,000) [32], [42], anti-Crp polyclonal antiserum (1∶5,000) [43], anti-GFP monoclonal antiserum (1∶5,000, Roche). Anti-rabbit horseradish peroxidase-conjugate was used as the secondary antibody at a final dilution of 1∶20,000. The ECL+ chemiluminescence system was used for the detection (GE Healthcare Life Sciences).

### Hemolytic activity assay

Contact hemolytic assay was performed as described earlier [38], [44]. Briefly, rabbit red blood cells (5%) were suspended in PBS containing 0.1% gelatin. One hundred µl of the blood suspension was loaded into the wells of a 96-well microtiter plate containing 50 µl of serially diluted OMV preparations or purified VCC. The microtiter plate was incubated at 37°C for 120 min and subsequently centrifuged at 1500×*g* for 10 min. One hundred µl from each well was transferred to a new plate and the absorbance at 540 nm was measured. The percent hemolysis was calculated as: hemoglobin released from OMV-treated RBCs/hemoglobin released from 1% Triton X-100-treated RBCs.

### Autophagy analysis by immunoblotting and confocal microscopy

For monitoring autophagy, 1×10^5^ HEK293 cells were seeded in 24-well culture plates (Thermo Scientific Nunclon) and onto glass coverslips overnight. Cells were incubated with OMVs or purified VCC for 6 h and 20 mM Tris-HCl pH 8.0 buffer was used as a negative control. For microscopic analysis, after the incubation, cells were fixed with 2% paraformaldehyde in PBS (pH 7.3) for 10 min. Fixed cells were washed with PBS and subsequently incubated with 0.1 M glycine and permeabilized with 0.5% Triton X-100. Nuclei were stained with DAPI. The samples were mounted on slides with Dako fluorescent mounting media and analyzed using Nikon D-Eclipse C1 Confocal Laser with a NIKON Eclipse 90i microscope and EZ-C1 3.80 imaging software. Images were taken with a Plan Apo NIKON 60X objective. For immunoblotting, all cells were harvested and pellets were lysed and suspended with dithiothreitol (DTT), 1X PBS, and Laemmli sample buffer. LC3 processing and GFP cleavage were analyzed using anti-GFP (Roche) monoclonal antibody (1∶5,000); band density was quantified by Quantity One 1-D analysis software.

### Expression of *V. cholerae* cytolysin in *E. coli*

*V. cholerae* cytolysin (*vcc*) gene was expressed in *E. coli* strain MC1061 by transforming *E. coli* competent cells with a plasmid carrying a *vcc* clone. In order to clone the *vcc* gene with its native promoter, a *V. cholerae* genomic library was constructed as described elsewhere [45]. Briefly, *V. cholerae* genomic DNA was isolated using (QIAamp DNA Mini Kit, Qiagen). Two µg of purified DNA was partially digested using HindIII and resolved on an agarose gel to obtain 4- to 6-kb fragments. Purified DNA fragments were ligated to similarly digested pBR322 vector. The resulting genomic library was propagated by transforming the ligation mixture into electro-competent *E. coli* DH5α cells. Ampicillin-resistant transformants were selected and screened for hemolysis on blood agar plates. Positive hemolysis transformants were then sub-cultured to isolate plasmid. After confirming the presence of the *vcc* gene by PCR, a plasmid clone denoted pBAB2 was used to overexpress VCC in the *E. coli* strain MC1061.

## Results

### Periplasmic localization of VCC during transport through the periplasm

**Figure 1:**
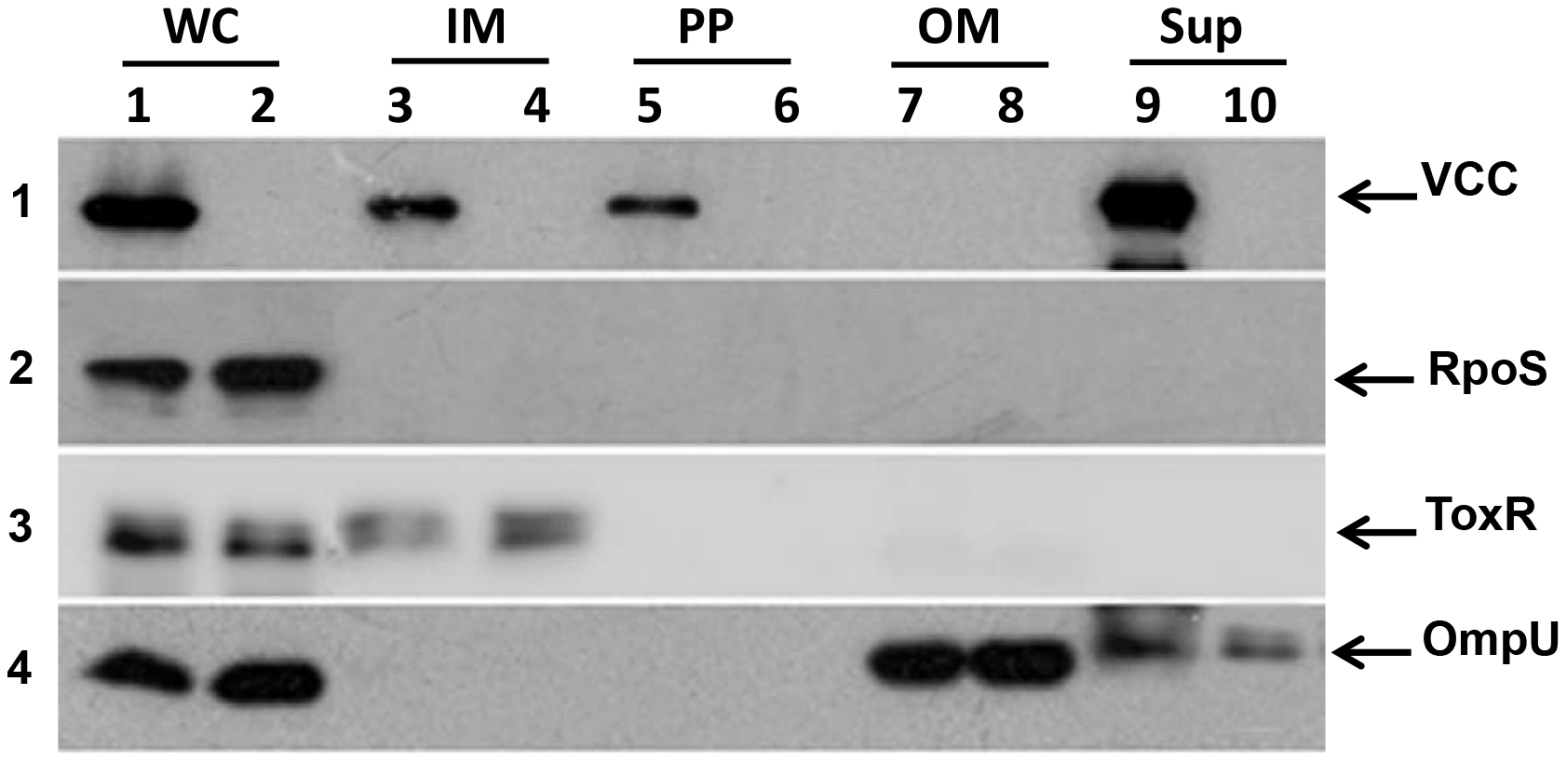
Immunoblot analysis of cell fractions. (A) Whole cell lysate (WC), Inner membrane (IM), periplasmic (PP), outer membrane (OM) fractions and culture supernatant (Sup) from the NOVC strain V:5/04 (lanes 1, 3, 5, 7, 9) and its isogenic Δ*vcc* mutant (MDS-1) (lanes 2, 4, 6, 8, 10), respectively. The samples were subjected to immunoblot analysis to detect VCC and different internal controls to rule out cross-contamination during fractionation; RpoS (as a cytoplasmic marker, panel 2), ToxR (as a inner membrane marker, panel 3), and OmpU (as an outer membrane marker, panel 4).

To investigate the sub-cellular localization of VCC, the NOVC strain V:5/04 was grown to OD_600 nm_ = 2.0. The bacteria were harvested and sub-cellular fractionation was performed as described in materials and methods. The whole cell lysate (WC), Inner membrane (IM), periplasmic (PP), outer membrane (OM) fractions and culture supernatant (Sup) were subjected to SDS-PAGE and immunoblot analysis using anti-VCC antiserum. A protein band corresponding to VCC was observed in WC, IM, and PP fractions (Fig. 1, panel 1; lanes, 1, 3, and 5; Fig. S1, panel 1). In addition, VCC was efficiently secreted from the bacterial cells into the culture supernatant (Fig. 1, panel 1; lane 9). The periplasmic fraction was analyzed by immunoblot using a periplasmic protein marker, β-lactamase. As shown in (Fig. S1, panel 4), the β-lactamase protein was clearly detected in the periplasmic fraction. To rule out any possibility of contamination of other cellular fractions in the PP fractions, the fractionation samples were further subjected to immunoblot analysis using antisera against internal control proteins such as either RpoS (Fig. 1, panel 2) or cAMP-receptor protein (Crp) as a cytoplasmic marker Fig. S1, panel 2), inner membrane-bound regulator ToxR as an inner membrane marker (Fig. 1, panel 3; Fig. S1, panel 3), and OmpU as an outer membrane marker (Fig. 1, panel 4; Fig. S1, panel 5). We found no trace of non-periplasmic cellular proteins in the periplasmic fractions. Taken together, these results indicated that VCC is a secreted protein that can localize in the periplasm during export to the extracellular space.

### Release of VCC in association with OMVs from non-O1 non-O139 *V. cholerae*

**Figure 2:**
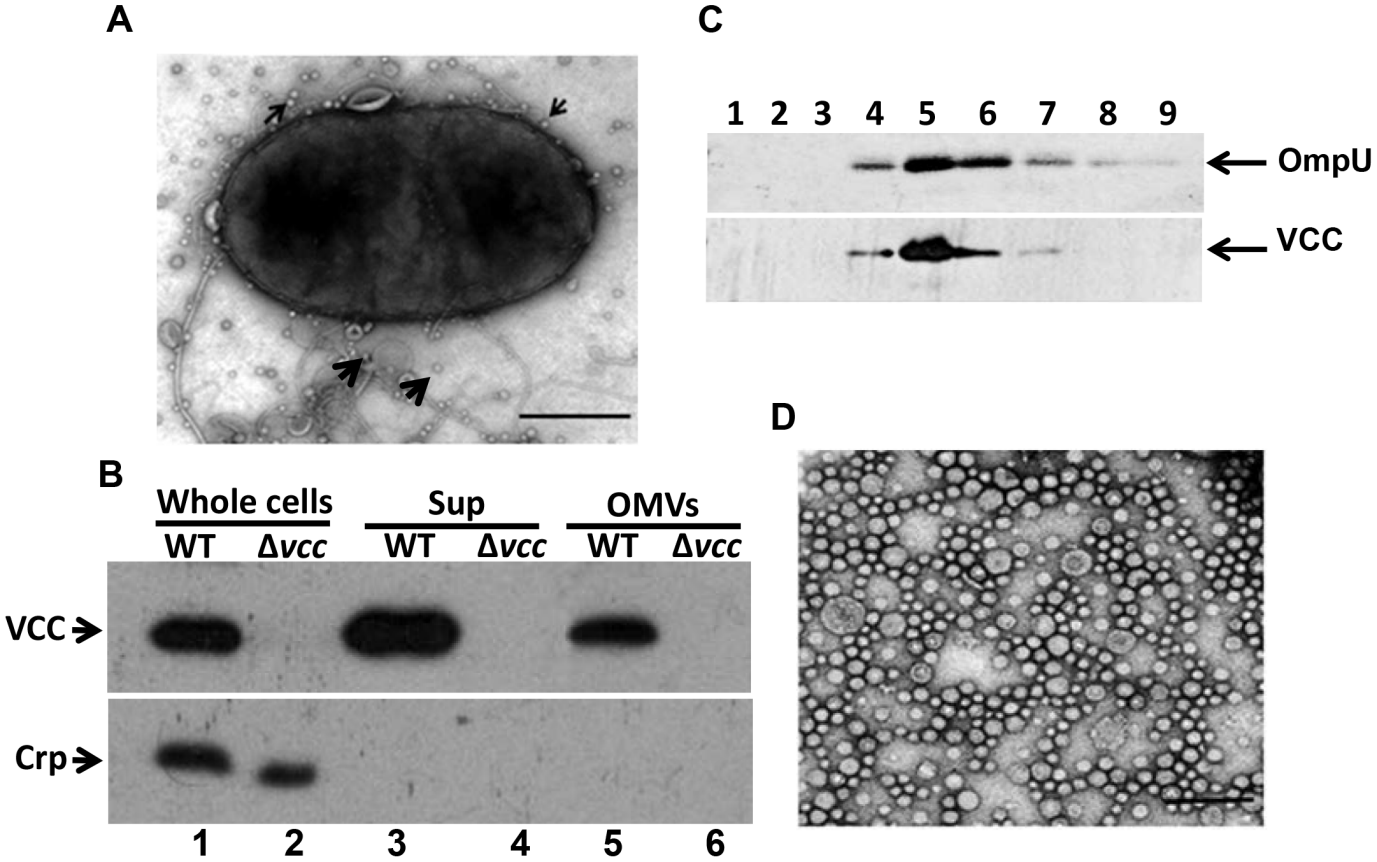
Ultrastructural and immunoblot analyses of OMVs. (A) Electron micrograph of a bacterial cell. OMVs released from the surface of the bacterial cell are shown (arrows). Bar = 200 nm. (B) Upper panel: Immunoblot analysis of the VCC in whole cell lysate (lanes 1, 2), supernatant [sup (lanes 3, 4)], and outer membrane vesicles [OMVs (lanes 5, 6)] from NOVC strain, V:5/04 (lanes 1, 3, 5) and its *vcc* mutant (lanes 2, 4, 6), respectively. Lower panel: A cytosolic protein (Crp) was used as an internal control. (C) Immunoblot analysis of protein fractions obtained by Optiprep density gradient centrifugation. Lanes 1–9 represents fractions 4–12 respectively. The arrows show OmpU and the VCC protein immunoreactive bands. (D) Electron micrograph of a purified OMV sample. Negative staining of the sample was performed as described in the material and methods. Different OMV sizes were observed by electron microscopy. Bar = 200 nm.

*V. cholerae* produces and secretes a variety of toxins into the extracellular environment [2]. It was reported that cholera-causing *V. cholerae* strains release abundant vesicles during their normal course of growth [46], [47]. To investigate the release of OMVs from non-cholera-causing NOVC strains and virulence factors associated with these OMVs, we isolated OMVs from the NOVC strain V:5/04 and examined the OMV ultrastructures by transmission electron microscopy (Fig. 2). We observed several OMVs blebbing out from the surface of a bacterial cell (Fig. 2A). We isolated OMVs from the V:5/04 culture using a sequential centrifugation method, as described in the materials and methods, to obtain a crude OMV sample. Subsequently, the crude OMV sample from strain V:5/04 was fractionated using optiprep density gradient centrifugation. Fractions 1–9 from the gradient centrifugation were analyzed by immunoblot. The OmpU protein was used as a marker for detection of OMVs as OmpU is a major outer membrane protein of *V. cholerae.* As shown in Fig. 2C (upper panel), OmpU immunoreactive bands were observed in fractions 4–7. We selected fraction 5, which showed the highest reactivity, for further experiments. First, we examined fraction 5 by electron microscopy using negative staining as described in the materials and methods. As shown in Fig. 2D, we observed OMVs with diameters ranging from 25–50 nm. OMVs are known to function as vehicles to transport periplasmic contents by enclosing these contents into the lumen of the OMVs [28]. OMVs isolated from culture supernatants of the VCC-producing strain V:5/04 and the isogenic *vcc* mutant (MDS-1) were analyzed by immunoblot using anti-VCC antiserum as described in the materials and methods. VCC was secreted in association with OMVs from the wild type *V. cholerae* strain V:5/04 (Fig. 2B, upper panel, lane 5). In order to analyse how much percentage of total secreted VCC was released in association with OMVs, we performed immunoblot analysis of total secreted VCC, non-vesicle associated VCC (VCC in the supernatant after ultracentrifugation), and OMV-associated VCC (VCC in the pellet after ultracentrifugation) (Fig. S2A, upper panel). The percentage of OMV-associated VCC was analysed semi-quantitatively using a Fluor-S Multi-Imager (Bio-Rad). The densitometric quantification of supernatant and OMVs samples showed that approximately 45% of the VCC was associated with OMVs (Fig. S2B, panel 3). There was no cytoplasmic marker protein, Crp, in the OMV sample (Fig. 2B, lower panel, lane 5), indicating that the presence of VCC in the OMVs was not a consequence of contamination by the cytosol due to bacterial cell lysis. The immunoblot membrane that was used to probe OMV membrane fractions with anti-OmpU antiserum was stripped and re-probed with anti-VCC antiserum, and as shown in Fig. 2C, lower panel, the VCC protein was detected in the same fractions where the OmpU protein also was detected. Taken together, these results demonstrated that the VCC protein from *V. cholerae* strain V:5/04 was secreted in association with OMVs.

### Tight association of VCC in the OMVs of *V. cholerae* strain V:5/04

**Figure 3:**
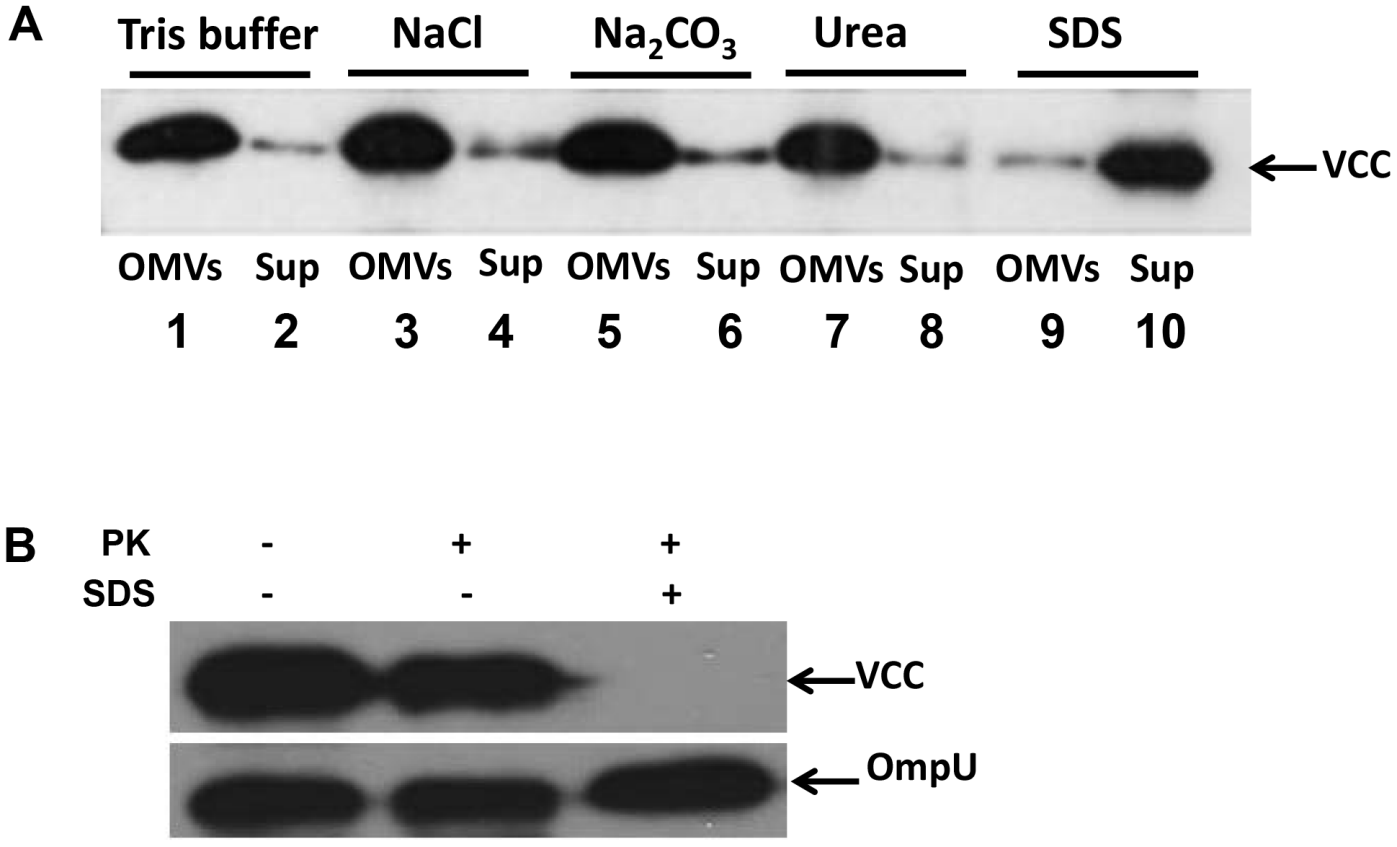
VCC is tightly associated with NOVC OMVs. (A) Dissociation assays using OMVs from the NOVC strain V:5/04. Samples of OMVs in 20 mM Tris-HCl pH 8.0 were treated for 60 min on ice in the presence of: 20 mM Tris-HCl pH 8.0 (buffer), NaCl (1 M), Na_2_CO_3_ (0.1 M), urea (0.8 M), SDS (1%), respectively. Samples were then centrifuged and the resulting OMVs (lanes 1, 3, 5, 7, 9) and Sup (lanes 2, 4 6, 8, 10) were analyzed by immunoblot using anti-VCC antibody. (B) Proteinase K protection assay. An equal amount of vesicles from the wild type NOVC strain V:5/04 was treated with 1.0 µg ml^−1^ of proteinase K (PK) in the presence or absence of 1% SDS. Samples were subjected to immunoblot analysis using anti-VCC antiserum and the OmpU protein (detected by anti-OmpU antiserum) was used as internal control.

During formation of OMVs periplasmic proteins may be included as cargo proteins internally in their lumen. However, a secreted protein can also tangentially or covalently attach to outer surface. To further investigate the nature of the association between OMVs and VCC, we performed a dissociation assay as described in the materials and methods. The VCC protein was recovered with OMVs in the pellet after treatment with NaCl, Na_2_CO_3_, or urea (Fig. 3, lanes 3, 5, and 7), as well as after washing with Tris buffer, which served as a control (Fig. 3, lane 1). However, when the OMV samples were treated with 1% SDS, the VCC protein could not be detected in the pellet with the OMVs, but instead was released and remained in the supernatant after subsequent centrifugation (Fig. 3, lane 10). These results suggested that VCC was tightly associated with the OMVs. Resistance to a high concentration of urea and liberation after SDS solubilization, as well as localization of VCC in the periplasmic space of bacterial cells indicated that the protein was not merely present as a protein aggregate that fortuitously had been pelleted with OMVs during the isolation procedure. We suggest that VCC most likely became located inside the OMVs during their formation and release from the bacterial cells.

To further assess how VCC is present in OMVs we tested the proteinase K susceptibility of OMV-associated VCC as described in the materials and methods. When OMVs from the wild type strain, V:5/04, were incubated with proteinase K in the presence of SDS, clear proteolytic digestion of the VCC was detected but no significant digestion occurred in the absence of the outer membrane damaging agents, indicating that the VCC was protected by the vesicle architecture (Fig. 3B).

**Figure 4:**
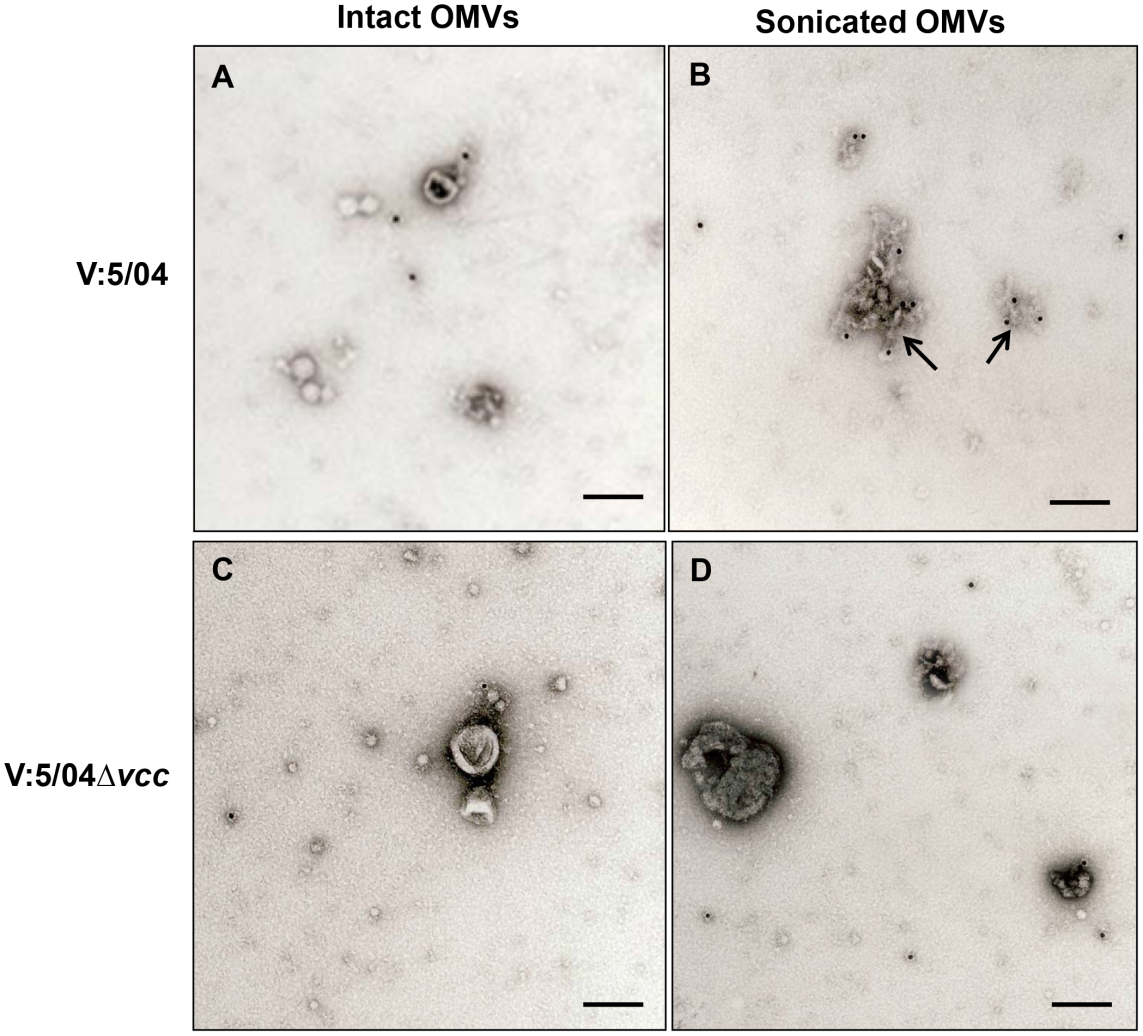
Immunoelectron microscopic detection of VCC in OMVs. Immunogold-labelling was performed with OMVs from wild type NOVC strain V:5/04 (A–B) and the Δ*vcc* mutant (C–D). Tests were done both with intact vesicles (A, C) and with vesicles ruptured by sonication (B, D). Arrows indicate gold particles associated with OMVs. Bars; 100 nm.

To more directly monitor the presence of VCC inside the OMVs, we performed immunoelectron microscopic analyses using anti-VCC antiserum in the immunogold labeling method. We detected the deposition of gold particles mainly on the material of the ruptured vesicles from wild type *V. cholerae* strain V:5/04 (Fig. 4B) whereas there were no gold particles observed in association with un-ruptured OMVs from the wild type strain V:5/04 (Fig. 4A) or the ruptured OMVs from the Δ*vcc* mutant (Fig. 4D). It appeared that due to the rupture of the OMVs the VCC was accessible to the immunogold probe. The results strongly support the suggestion that the VCC protein was associated with OMVs of *V. cholerae* strain V:5/04 and it appeared that the protein was internal to the vesicle membrane.

**Figure 5:**
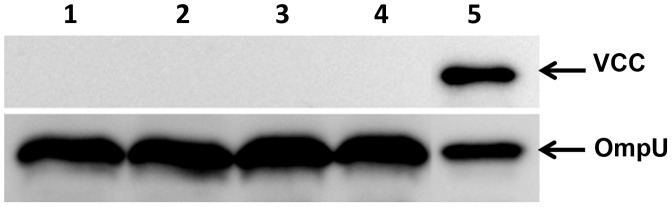
Re-association tests with VCC and OMVs. Immunoblot analysis using anti-VCC antiserum (upper panel) and anti-OmpU antiserum (lower panel). OmpU was used as an internal control to show the presence of OMVs in the samples. Samples with equal amounts of OMVs from the Δ*vcc* mutant were incubated with PBS (lane 1), with 0.6 nM of purified VCC (lane 2), with vesicle free supernatant from the wild type strain V:5/04 (lane 3), with vesicle free supernatant from the V:5/04Δ*vcc* strain (lane 4). V:5/04 OMVs was used as a positive control sample (lane 5).

The results described above prompted us to investigate if free secreted VCC or purified VCC can re-associate on the surface of the OMVs. In order to test this possibility, a re-association test was performed that included immunoblot analysis using anti-VCC antiserum as described in the materials and methods. In this experiment, we used purified VCC protein and a vesicle free culture supernatant of *V. cholerae* strain V:5/04 to test if either of these VCC protein preparations could attach or re-associate on the surface of the OMVs isolated from the Δ*vcc* mutant. As shown in Fig. 5, (upper panel), no VCC protein was detected in the OMV samples treated with PBS, with purified VCC, with OMV-free culture supernatant of the wild type strain V:5/04, or with OMV-free culture supernatant of the V:5/04Δ*vcc* mutant (Fig. 5, lanes 1–4). A sample of OMVs from the wild type *V. cholerae* strain V:5/04 was included as positive control (Fig. 5, lane 5). To verify the presence of OMVs in the different samples, immunoblot analysis using anti-OmpU antiserum was performed (Fig. 5, lower panel). The test did not lend support to the possibility that free soluble VCC readily would associate on the surface of the OMVs. Taken together, our results suggest that the presence of VCC in the OMVs from the wild type *V. cholerae* strain V:5/04 occurred as a result of localization during OMV formation and was not due to the binding of VCC on the surface of the OMVs or co-precipitation of VCC during the vesicle isolation procedure.

### Vesicle-associated VCC is biologically more active than pure VCC

**Figure 6:**
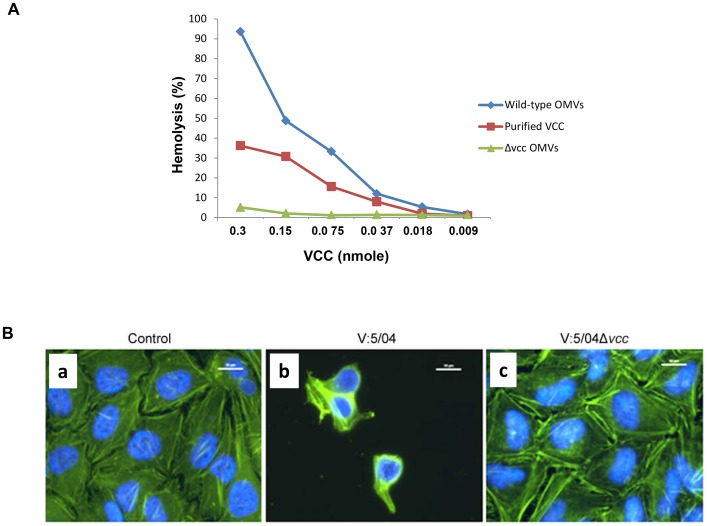
Hemolytic and cytotoxic effects of OMV-associated VCC. (A) Quantification of hemolytic activity of VCC in OMVs and comparison with activity of purified VCC. Contact hemolytic assay using 5% rabbit red blood cells was performed as described in the materials and methods. (B) Cytotoxic effect on HeLa cells (a) Control cells were treated with Tris buffer; (b) cells were treated with OMVs from wild type NOVC strain V:5/04; (c) cells were treated with OMVs from the Δ*vcc* mutant. After 6 h the HeLa cells were fixed, permeabilized, and then subjected to staining as described in the materials and methods. Staining, actin filament (green) and nucleus (blue). Magnification, ×1000. Bars = 10 µm.

To determine the biological activity of OMV-associated VCC, we performed contact hemolytic activity assays (Fig. 6A) and cell cytotoxicity assays (Fig. 6B). The relative hemolytic activity against red blood cells was tested by measuring the release of hemoglobin after co-incubation of 5% rabbit red blood cells with OMVs. In order to compare the hemolytic activity between the OMV-associated VCC and puried VCC, we first estimated the amount of VCC in the OMVs by comparing the immunoreaction density of OMV-associated VCC with that of known amounts of purified VCC. We found that 100 µl of our OMVs samples from the wild type *V. cholerae* strain V:5/04 contained an amount of VCC protein that was equivalent to 0.3 nmole of purified VCC. Then we compared the hemolytic activity of the VCC in OMVs with that of the purified VCC. As shown in Fig. 6A, the two-fold serial dilution of both purified VCC and of OMVs from the wild type strain V:5/04 elicited dose-dependent hemolysis of the blood cells. Moreover, at each of the tested dilutions, the VCC activity of OMVs was consistently higher than that of the corresponding amount of purified VCC.

To assay the cytotoxic effect on growing target cells, cultured HeLa cells were treated with OMV samples and examined by microscopic analysis. OMVs from the wild type NOVC strain V:5/04 showed a clear cytotoxic effect on HeLa cells and due to detachment and lysis few cells remained after the 6 h duration of the treatment (Fig. 6B, panel b), whereas there was no obvious cytotoxic effect during treatment with OMVs isolated from the Δ*vcc* mutant (Fig. 6B, panel c).

### OMV-associated VCC can induce autophagy in HEK293 cells

Increasing evidence suggests a possible link between autophagy induction and bacterial toxins [48]. Recent work on VCC indicated that autophagy may play a role in cellular defense against the VCC toxin [22]. Autophagosome formation is a critical step in autophagy. The process starts with extension of a free membrane around a target cellular organelle and surrounding cytosolic proteins, leading to the formation of an autophagosome. Simultaneously, the cytosolic form of microtubule-associated protein 1A/1B-light chain 3 (LC3-I) undergoes conjugation with phosphatidylethanolamine (PE) to form a lipidated derivative of LC3 (LC3-II). LC3-II relocates almost exclusively to the autophagosome membrane until it is delivered to the lysosome for degradation in the autolysosome. Therefore, monitoring lysosomal turnover of LC3-II effectively determines the degree of autophagy induction in cells [49].

**Figure 7:**
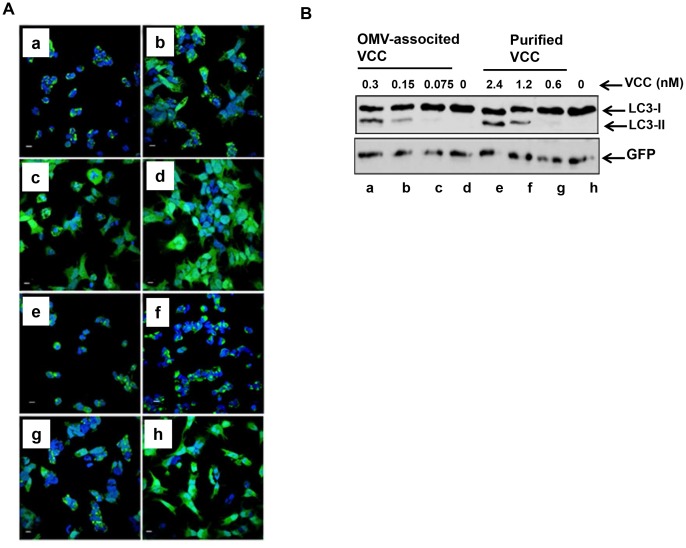
VCC induced autophagy. Confocal microscopy analysis (A) and immunoblot analysis (B) of autophagy induced by vesicle-associated VCC in HEK293 cells stably expressing a GFP::LC3 fusion protein. HEK293 cells were treated with: 0.3 nM, 0.15 nM, 0.075 nM of OMVs associated VCC (a, b, c); OMVs isolated from the Δ*vcc* mutant (d); 2.4 nM, 1.2 nM, and 0.6 nM of purified VCC respectively (e, f, g, h).

In our assay, HEK293 cells expressing GFP-LC3 were incubated with different amounts of OMVs from the wild type NOVC strain V:5/04 or its isogenic Δ*vcc* mutant (MDS-1) or with purified VCC for 6 h. Confocal microscopy and immunoblot analysis using anti-GFP antibody were used to monitor autophagy. Cells infected with 0.3 and 0.15 nM VCC containing OMVs isolated from the wild type strain V:5/04 showed several autophagosomes visualized as bright GFP-LC3 punctuated particles (Fig. 7A, panel a and b respectively) and showed clear processing of the LC3-I form into the LC3-II form (Fig. 7B, lanes a and b respectively) in a dose dependent manner. To obtain the same level of autophagy response, 2.4 and 1.2 nM of purified VCC was required (Fig. 7A, panels e and f; Fig. 7B, lanes e and f respectively). The autophagy response induced by the purified VCC was also in a dose dependent manner. The OMVs from the Δ*vcc* mutant was used as a negative control (Fig. 7A, panel d and Fig. 7B, lane d). Taken together, our results show that OMV-associated VCC is relatively more active than the purified VCC in different biological activities.

### Vesicle-mediated release of VCC is common among *V. cholerae* strains

**Figure 8:**
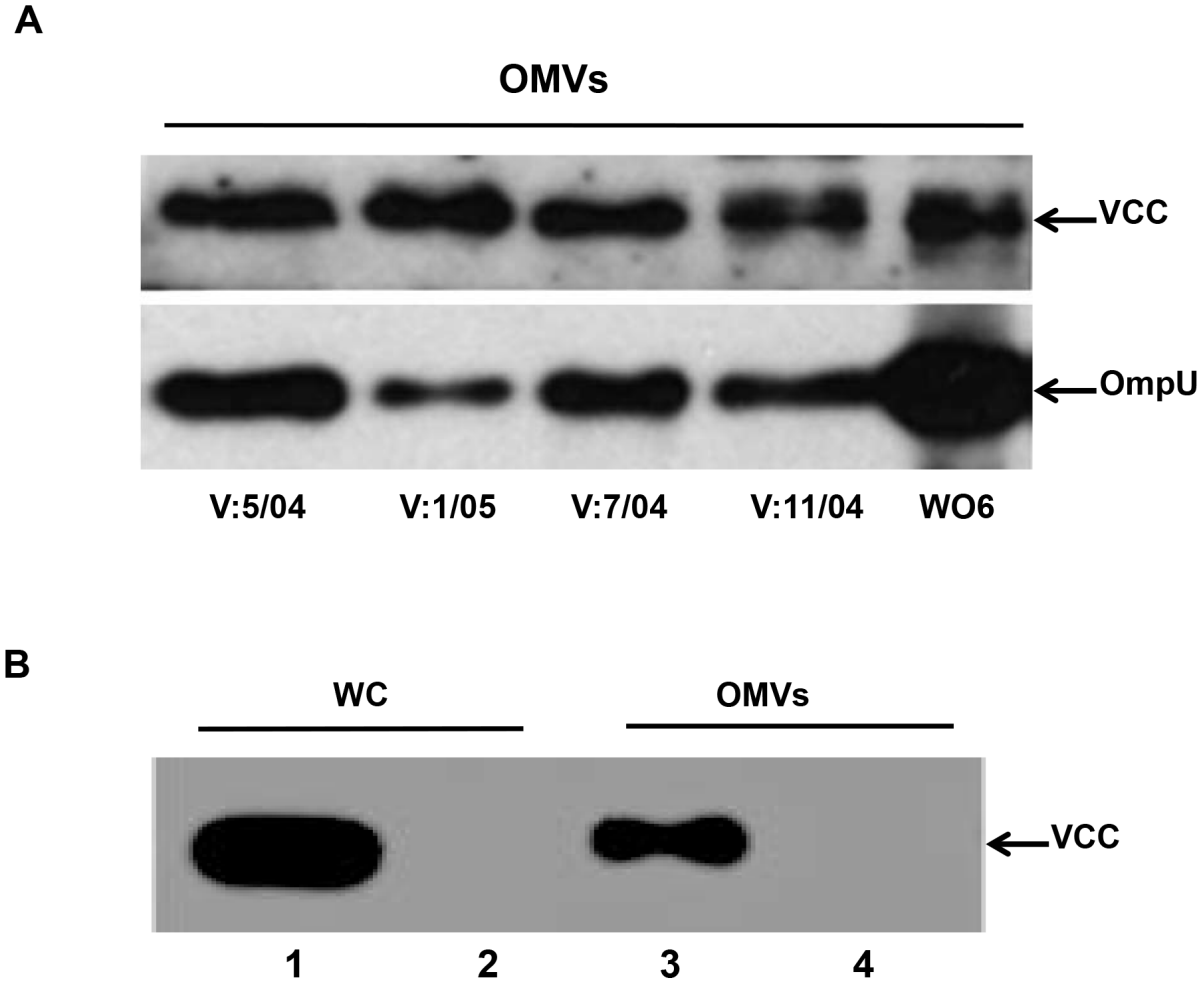
OMV-mediated transport of VCC is a common phenomenon. (A) Immunoblot analysis of OMVs from *V. cholerae* strains V:5/04, V:1/05, V:7/04,V:11/04 and WO6. (B) Immunoblot analysis of whole cell lysate (WC) and OMVs of *E. coli* strains MC1061/pBAB2 (1, 3) and MC1061/pBR322 (2, 4), respectively.

To determine whether vesicle-mediated transport of VCC is specific to the V:5/04 strain or is a common feature among *V.* *cholerae* strains, we analyzed OMVs isolated from different *V.* *cholerae* strains. Immunoblot analysis revealed the presence of VCC in samples of OMVs from strains of different serogroups, strongly suggesting that vesicle-mediated release of VCC is not strain or serogroup-specific but is a common phenomenon in *V.* *cholerae* (Fig. 8A). To investigate if OMV-associated VCC secretion occurs when the *vcc* gene is heterologously expressed in *E. coli*, the *vcc* gene was cloned and introduced into the *E. coli* K-12 strain MC1061. OMVs from these *E. coli* derivatives were isolated and tested for the presence of VCC using anti-VCC antiserum. As shown in Fig. 8B, lane 3, an abundant amount of VCC protein was detected in association with OMVs isolated from *E. coli*. We suggest that OMV-mediated release of VCC is a general phenotype.

## Discussion

VCC is a pore-forming toxin that induces lysis of different mammalian cells by forming transmembrane 14-stranded β-barrel channels in the plasma membrane [50]. At sub-lytic concentration, VCC can trigger vacuolization and apoptosis of epithelial and immune cells [14], [51]. It also can enhance the virulence efficiency of *V. cholerae* [2]. In addition, there are several studies on characterization of VCC regarding biophysical activities, including carbohydrate binding activities and membrane targeting domain analyses, however, there has been no detailed analysis of VCC secretion from the bacterial cells [52]. VCC is known to be an efficiently secreted protein, but the specific *V. cholerae* secretion pathway(s) and cellular machinery involved are only poorly understood. Our present study described sub-cellular localization of *V. cholerae* toxin VCC, and characterized a final step in its extracellular transport. The secretion system of another pore-forming toxin, α-hemolysin (HlyA) of *E. coli,* has been analyzed in detail by several groups that have described its translocation through the type I secretion system (T1SS) [53]. The T1SS employs a trans-envelope apparatus that delivers proteins directly to the extracellular space, without periplasmic intermediates. On the other hand, secretion of another pore-forming toxin from *Vibrio vulnificus*, hemolysin (Vvh), occurs via the type II secretion system (T2SS), a two-step pathway in which the protein initially translocates to the periplasm, and is then transported into the extracellular space.

Given the structural and functional homology between VCC and Vvh and the presence of an N-terminal signal sequence, we suspected a possible role for T2SS in VCC secretion. Moreover, a recent study involving secretome analysis using sequential isotope tagging for relative and absolute quantification (iTRAQ), liquid chromatography and mass spectroscopy techniques identified the relative absence of VCC in the *V. cholerae* T2SS mutants Δ*eps and* Δ*epsD*, which further suggested a role for T2SS in VCC secretion [54]. Since T2SS essentially involves an intermediate periplasmic localization of its cargo proteins, we analyzed the presence of VCC in different cellular compartments of *V. cholerae* using a cell fractionation protocol and tested the route of VCC secretion. We observed that VCC was present in periplasmic fraction indicating that it represents an intermediate step during its secretion outside the bacterial cells. Taken together, these data suggest that VCC of the *V. cholerae* NOVC strain V:5/04 is secreted via the inner membrane using the T2SS machinery as a first step and, subsequently, from the periplasmic space some VCC can be released in association with OMVs.

The release of OMVs is a common phenomenon of Gram-negative bacteria during their normal course of growth and metabolic activity [46], [55]. OMVs enclose the different periplasmic proteins more or less efficiently into the lumen during vesicle formation [56], [57]. There are several reports for *E. coli*, *Campylobacter jejuni, Legionella pneumophila*, and *Helicobacter pylori* of bacterial virulence factors that are transported to the extracellular environment using OMVs as a delivery vehicle [26], [28], [31], [38], [58]. In this study, we demonstrated that the VCC was merely present in the lumen of OMVs and we suggest that the vesicle architecture somehow protects the VCC from degradation. A general phenomenon of pore-forming toxins is their speedy and irreversible loss of biological activity in vitro, which may be due to self-aggregation and/or premature oligomerization. OMVs might play a role in the prolongation of biological activity of their luminal toxins by protecting them from rapid self-aggregation [59]–[61]. Our analysis with biological assays of OMVs from the *V. cholerae* strain V:5/04 confirmed that OMV-associated VCC is indeed biologically active and induces toxic effects on mammalian cells in a dose-dependent manner. Interestingly, vesicle associated VCC was biologically more active in comparison with similar amounts of purified VCC. Furthermore, analysis of both environmental and clinical isolates of different *V. cholerae* serogroups demonstrated that the transport of VCC by OMVs is a common feature among *V. cholerae* strains.

In recent years, several lines of evidence have indicated the important role played by autophagy in numerous physiological and pathological processes, including bacterial infections [62]. Recently, it was suggested that autophagy might have dual roles during bacterial infection [63]. Canonical autophagy limits bacterial replication, and degradation occurs upon fusion with a lysosome. However, autophagy can have a pro-bacterial effect, mainly by inhibiting lysosomal degradation. Our studies with VCC-associated OMVs indicate that the toxin triggers an autophagic response in the target cell and, more importantly, demonstrated for the first time that autophagy operates as a cellular defense mechanism against an OMV-associated bacterial virulence factor. However, further research is required to elucidate mechanisms regulating the interplay between autophagy and secreted OMV-associated bacterial proteins, as well as signaling pathways involved in autophagy induction. Understanding how OMV-associated bacterial pore-forming toxins modulate autophagy may potentially contribute to development of new anti-microbial strategies and therapeutics.

## Supporting Information

Figure S1 **Periplasmic localization of VCC.** Immunoblot analysis of whole cell lysate (WC) and periplasmic (PP) fractions from V:5/04 strain carrying the β-lactamase expressing plasmid pMMB66EH. The samples were subjected to immunoblot analysis to detect VCC and different internal controls to rule out cross-contamination during fractionation; Crp (as a cytoplasmic marker, panel 2), ToxR (as an inner membrane marker, panel 3), β-lactamase (as a periplasmic marker, panel 4) and OmpU (as an outer membrane marker, panel 5).
(TIF)

Figure S2 **Estimation of amount of OMV associated VCC.** (A) Immunoblot analysis of the total secreted VCC using anti-VCC antiserum (lane 1), supernatant after removal of OMV samples (lane 2), and OMVs (lane 3) (upper panel); OmpU detected by anti-OmpU antiserum was used as an internal control for the OMV samples (lower panel). All samples were concentrated 10 times relative to the culture volume. (B) Densitometry analysis of percentage of released VCC from the bacterial cells. Percentage of total secreted VCC (column 1; 100%), free soluble VCC in the culture supernatant after the removal of OMV samples (column 2; 55%), and OMV-associated VCC (column 3; 45%).
(TIF)
