# Supplementary figures and images for: Outer Membrane Vesicles Mediate Transport of Biologically Active Vibrio cholerae Cytolysin (VCC) from V. cholerae Strains
Source: PLoS One. 2014 Sep 4;9(9):e106731. doi: 10.1371/journal.pone.0106731 (PMC4154730; doi:10.1371/journal.pone.0106731)

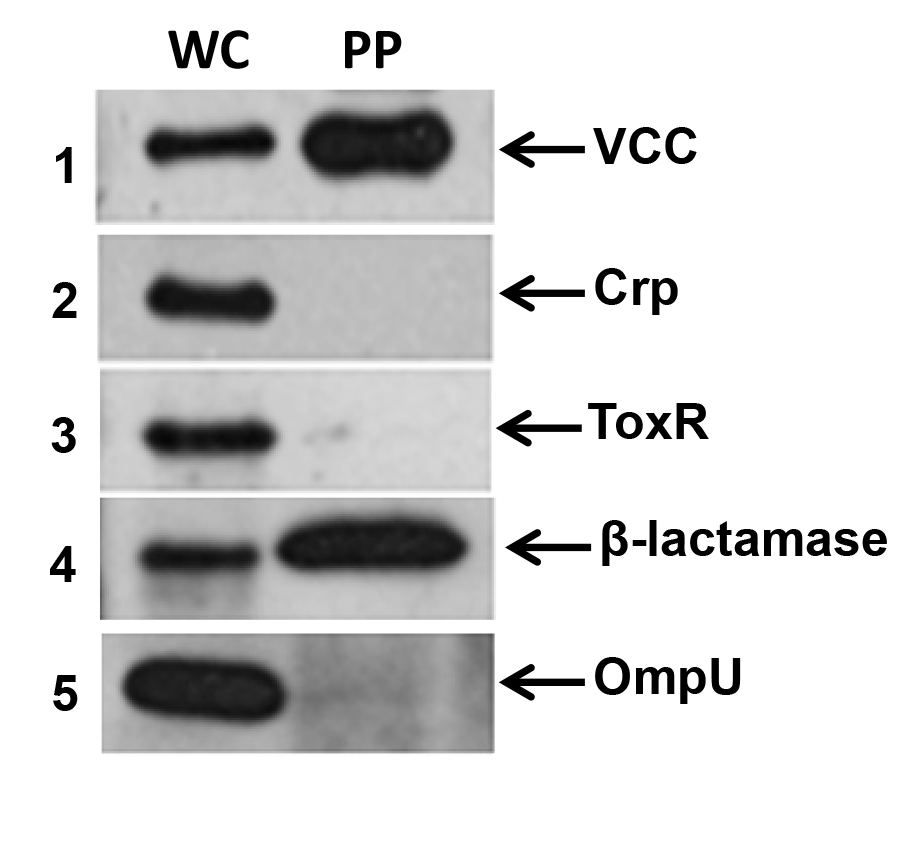

Supplement: Figure S1 — Periplasmic localization of VCC. Immunoblot analysis of whole cell lysate (WC) and periplasmic (PP) fractions from V:5/04 strain carrying the β-lactamase expressing plasmid pMMB66EH. The samples were subjected to immunoblot analysis to detect VCC and different internal controls to rule out cross-contamination during fractionation; Crp (as a cytoplasmic marker, panel 2), ToxR (as an inner membrane marker, panel 3), β-lactamase (as a periplasmic marker, panel 4) and OmpU (as an outer membrane marker, panel 5). (TIF) [file pone.0106731.s001.tif]

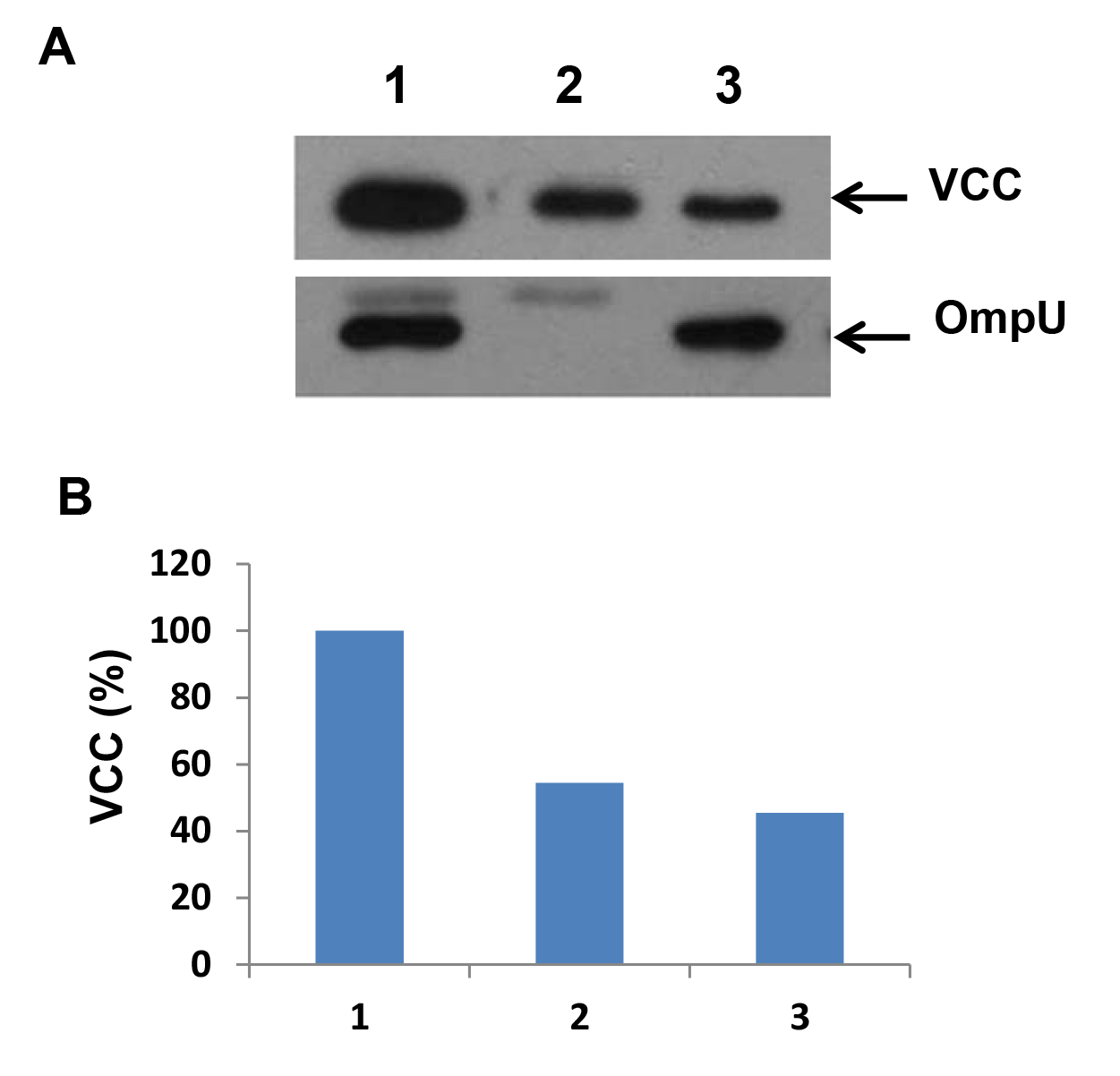

Supplement: Figure S2 — Estimation of amount of OMV associated VCC. (A) Immunoblot analysis of the total secreted VCC using anti-VCC antiserum (lane 1), supernatant after removal of OMV samples (lane 2), and OMVs (lane 3) (upper panel); OmpU detected by anti-OmpU antiserum was used as an internal control for the OMV samples (lower panel). All samples were concentrated 10 times relative to the culture volume. (B) Densitometry analysis of percentage of released VCC from the bacterial cells. Percentage of total secreted VCC (column 1; 100%), free soluble VCC in the culture supernatant after the removal of OMV samples (column 2; 55%), and OMV-associated VCC (column 3; 45%). (TIF) [file pone.0106731.s002.tif]
